# Marchiafava-Bignami disease mimics motor neuron disease: case report

**DOI:** 10.1186/1471-2377-13-208

**Published:** 2013-12-21

**Authors:** Yasunobu Hoshino, Yuji Ueno, Hideki Shimura, Nobukazu Miyamoto, Masao Watanabe, Nobutaka Hattori, Takao Urabe

**Affiliations:** 1 Department of Neurology, Juntendo University Urayasu Hospital, 2-1-1 Tomioka, Urayasu, Chiba 279-0021, Japan; 2 Department of Neurology, Juntendo University School of Medicine, 2-1-1 Hongo, Bunkyo, Tokyo 113-8421, Japan

**Keywords:** Marchiafava-Bignami disease, Motor neuron disease, Amyotrophic lateral sclerosis, Upper motor neuron signs, Lower motor neuron signs, Chronic alcoholism

## Abstract

**Background:**

Marchiafava-Bignami disease (MBD) is a rare neurologic complication of chronic alcohol consumption that is characterized by callosal lesions involving demyelination and necrosis. Various reversible neurologic symptoms are found in patients with MBD. Dysarthria and dysphagia are found in various neurological diseases.

**Case presentation:**

We report a 51-year-old man with chronic alcoholism and malnutrition who progressively developed dysarthria and dysphagia. On admission, the patient was alert with mild cognitive dysfunction. The facial expression was flat, and there was weakness of the orbicularis oris bilaterally. The patient’s speech was slurred, there was difficulty swallowing, and the gag reflex and palate elevation were poor. The jaw jerk reflex was brisk and the snout reflex was positive. Neither tongue atrophy nor fasciculation were found. Bilateral upper and lower limb weakness with increased bilateral upper limb reflexes and Babinski reflexes were found. Because he had progressive dysarthria and dysphagia with upper and lower motor neuron signs, the initial diagnosis was motor neuron disease. However, electrophysiological analysis was normal. The vitamin B1 level was 14 ng/mL (normal: >24 ng/mL), and MRI revealed hyperintense lesions in the splenium of the corpus callosum and the primary motor cortices bilaterally. After vitamin B therapy for 17 days, the neurological disorders alleviated concurrently with disappearance of the lesions on MRI, which led to the definitive diagnosis of MBD.

**Conclusions:**

MBD presenting with these lesions can mimic motor neuron disease clinically.

## Background

Marchiafava-Bignami disease (MBD) is a rare neurological disease related to chronic and heavy alcohol consumption and malnutrition, and is characterized by primary demyelination and necrosis of the central part of the corpus callosum [1-4]. The following pathognomonic MRI findings are critical for the diagnosis of MBD: hyperintense signal lesions without significant mass effect within the corpus callosum, which may extend to the genu and adjacent white matter on T2-weighted, fluid attenuated inversion recovery (FLAIR) and diffusion-weighted image (DWI) studies. To date, the pathologic mechanisms leading to MBD have not been fully elucidated [3,5-9]. Over 90% of the patients with MBD exhibited a poor prognosis [10]. However, MBD patients can recover completely with disappearance of the callosal and adjacent white matter lesions on serial MRI after adequate therapy in a few weeks to half a year [7,9,11,12]. MBD includes a variety of neurologic features such as seizures, confusion, and deterioration of consciousness, which can be difficult to differentiate from symptoms of other alcoholic neurological disorders [3]. Interhemispheric disconnection syndromes caused by disorders of the corpus callosum may be included in characteristic symptoms of the diagnosis of MBD [13,14].

Dysarthria and dysphagia can occur in various neurological disorders, including cerebrovascular disease, neurodegenerative disease, Guillain-Barré syndrome, and neoplastic disease [15-18], and are caused by disorders of cranial nerve motor nuclei in the lower brainstem resulting in lower motor neuron signs, as well as of the bilateral corticobulbar tracts resulting in upper motor neuron signs. In particular, progressive dysarthria and dysphagia are not infrequently found in patients with motor neuron disease (MND); 8% of patients with amyotrophic lateral sclerosis (ALS) present with progressive dysarthria and dysphagia as the initial symptoms [17].

Here we report a patient with a history of chronic alcoholism who developed progressive dysarthria and dysphagia. According to the mode of symptom onset and presenting neurological signs, the initial diagnosis was MND. However, an improvement of the neurological disorder concurrently with a change of MRI findings after therapy led to the definitive diagnosis of MBD.

## Case presentation

A 51-year-old man, with a 20-year history of heavy alcohol abuse (1.5 L of beer per day for 20 years often accompanied by 360 mL of shochu, a Japanese distilled spirit containing 25-35% alcohol by volume) and loss of appetite for 4 years, progressively developed slurred speech for 3 weeks. Subsequently, he choked while drinking and had difficulty swallowing food. Finally, he could not eat or drink, and was admitted to our department. He had previously been diagnosed with alcoholism and had a history of chronic obstructive pulmonary disease. At the age of 44, he underwent burr-hole drainage for bilateral chronic subdural hematomas. After surgery, he became independent regarding the activities of daily living. There was no family history of MND. On admission, blood pressure was 112/79 mmHg, and body height and weight were 183 cm and 48 kg, respectively. Neurological examination revealed an alert patient with a mini-mental status examination (MMSE) score of 22 points (orientation to time, -2 points; attention and calculation, -4; three word recall, -2). There was horizontal gaze paretic nystagmus bilaterally. The facial expression was flat, and there was weakness of the orbicularis oris bilaterally. Weakness of the frontalis muscle and orbicularis oculi was not found. The speech was slurred, and there was difficulty swallowing; the gag reflex and palate elevation were poor. The jaw jerk reflex was brisk and the snout reflex was positive. Emotional lability was not found. Neither tongue atrophy nor fasciculation were found. The tongue could be protruded from the mouth and remained midline, and moved adequately from side to side. Motor weakness was evident, with scores of 4 in the distal upper limbs bilaterally and 3 in the proximal lower limbs bilaterally on the Modified Medical Research Council’s manual muscle test (MMT). There was finger-to-nose incoordination bilaterally. The patient could not walk because of a wide-based gait with truncal instability. Deep tendon reflexes in the upper limbs were increased, and Babinski reflexes were positive bilaterally. Hoffmann reflexes and the forced grasp reflex were negative. The superficial vibratory and position senses were normal.

**Figure 1:**
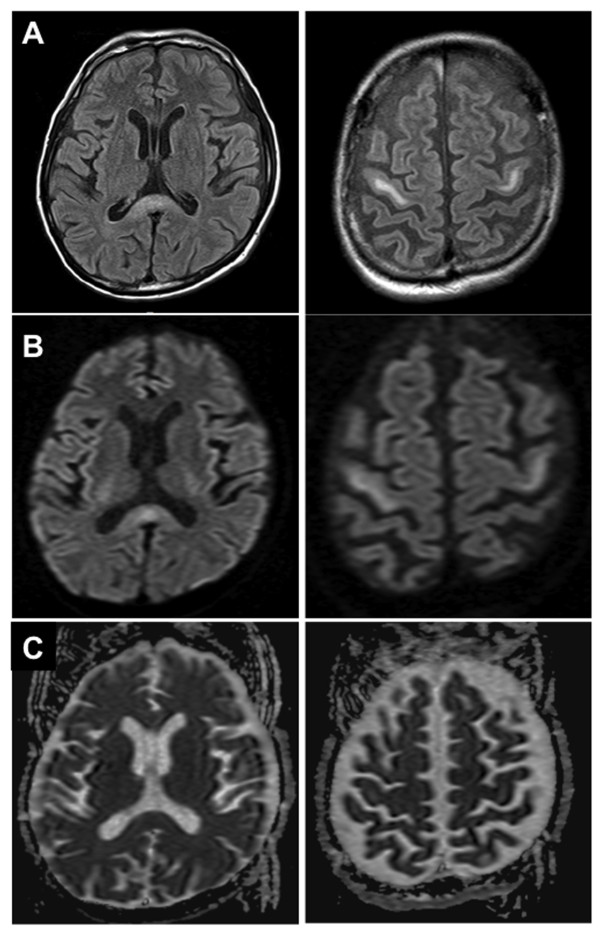
**Admission MRI.** Representative images of brain MRI on admission including fluid attenuated inversion recovery (FLAIR) **(A)**, diffusion-weighted image (DWI) **(B)**, and the apparent diffusion coefficient (ADC) map **(C)**, showing hyperintense lesions in the precentral gyrus bilaterally and the splenium of the corpus callosum.

The initial diagnosis was MND because of the development of progressive dysarthria and dysphagia with upper and lower motor neuron signs, and the limb weakness with upper motor neuron involvement. However, a nerve conduction study did not reveal prolonged distal latencies, conduction blocks, or an absent F-wave. Needle electromyography showed that normal unit potentials and no denervation potentials were found in the tongue, sternocleinomastoid, biceps or quadriceps femoris. Brain MRI demonstrated hyperintense lesions in the precentral gyrus bilaterally and in the splenium of the corpus callosum on FLAIR (Figure 1A). Those lesions were also hyperintense on DWI (Figure 1B), and the apparent diffusion coefficient (ADC) map did not show significant signal changes (Figure 1C). Laboratory test results included decreased vitamin B1 (14 ng/mL; normal: >24 ng/mL), and mild elevation of liver enzymes. The cerebrospinal fluid was normal.

**Figure 2:**
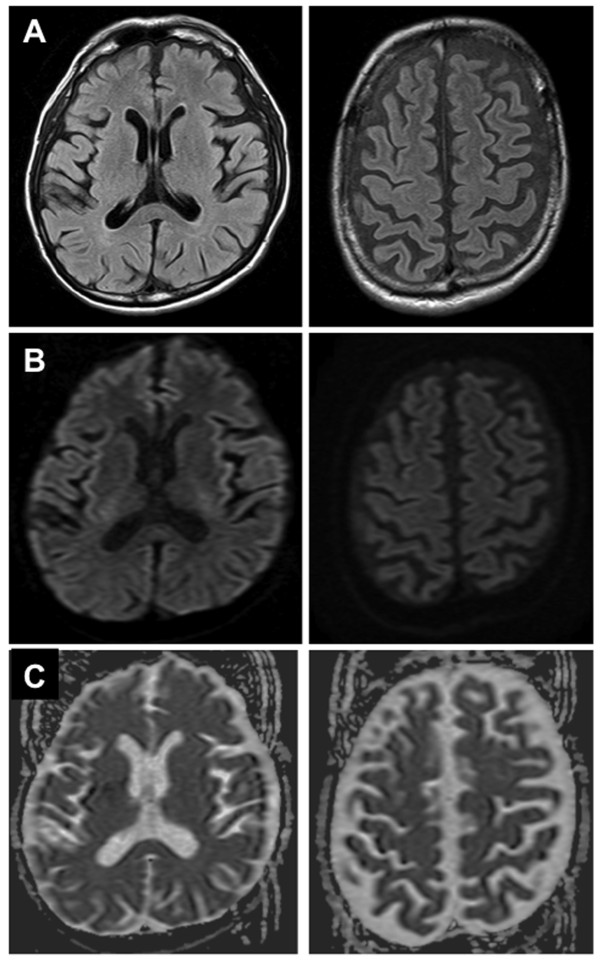
**MRI after therapy.** Representative FLAIR images **(A)**, DWI **(B)**, and ADC map **(C)** 17 days after admission, showing disappearance of the hyperintense lesions.

Complex vitamin B therapy, including 100 mg of thiamin, was started intravenously on the day of admission. After admission, the patient’s swallowing slowly improved, and gradually the speech became clear. On admission, only food of a pudding-like texture was tolerated, but 7 days after admission gruel-like foods were manageable, and 13 days after admission the patient was placed on a normal diet. On hospital day 14, the MMSE score had increased to 26 points (attention and calculation, -4), limb weakness had improved, and the patient could walk with a cane. Concurrently, hyperreflexia of the jaw jerk and bilateral upper limb reflexes were normalized, and the bilateral Babinski reflexes became negative. Gaze paretic nystagmus and finger-to-nose incoordination were also improved. Repeat MRI at 17 days after admission showed the disappearance of signal abnormalities in the splenium of the corpus callosum and the precentral gyrus on FLAIR and DWI (Figure 2A-C).

## Discussion

Specific clinical characteristics of the present case are the development of dysarthria and dysphagia with upper and lower neurons signs, and limb weakness with upper motor neuron signs. Disappearance of lesions in the corpus callosum and bilateral precentral gyrus was associated with an improvement of the clinical neurological disorders after vitamin B therapy.

MBD is most frequently seen in middle-aged or elderly chronic alcoholic males [6-9,11]. MBD was first reported in 1903 by Marchiafava and Bignami, who originally described the symptoms in Italian men with increased consumption of inexpensively manufactured Chianti red wine [1]. Currently, however, MBD is known to occur in patients with chronic consumption of other sorts of alcohol including whisky and French liqueur [6,7]. MBD has also been found in severely malnourished people without a history of alcoholism [7,18]. In the present case, long-term consumption of beer and Japanese distilled spirits and malnutrition might have been related to the pathogenesis of MBD. Although the precise mechanisms underlying development of MBD remain unknown, effects of toxic agents present in alcohol, vitamin B complex deficiency, or osmotic disorders have been considered as potential causes [3,4,7]. In a report of an MR spectroscopic study, it was suggested that inflammatory reactions accompanying demyelination and micronecrosis and secondary axonal damage might occur in the acute stage of MBD [19]. Presence of cerebral microhemorrhage on susceptibility-weighted imaging was reportedly associated with cognitive dysfunction in MBD patients, and cytotoxic edema on DWI and the ADC map might predict poor outcome [8,11].

The present patient progressively developed dysarthria and dysphagia in the setting of chronic alcoholism and malnutrition. In particular, the dysarthria included a poor gag reflex and palate elevation as lower motor neuron signs, and slurred speech, brisk jaw jerk reflex, and the snout reflex as upper motor neuron signs, which have been defined as mixed dysarthria that is not infrequently found in ALS [20]. Other neurological examinations revealed muscle weakness of the lower face and four limbs, as well as hyperreflexia in the upper limbs bilaterally and positive Babinski reflexes bilaterally. Thus, the clinical presentation mimicked MND, especially ALS. Interestingly, recent studies have shown that hyperintense signal lesions on FLAIR of the subcortical precentral gyrus bilaterally, as seen in the present case, were consistent features of ALS, and that the corpus callosum was also involved in the pathogenesis of ALS [21-23]. After vitamin B therapy, the current patient’s neurological disorders were alleviated concurrently with the disappearance of the lesions on MRI. This suggests that MBD with lesions of the bilateral precentral gyrus and splenium of the corpus callosum may cause progressive dysarthria and dysphagia with upper and lower neuron signs mimicking MND, symptoms that are improved by vitamin B therapy. To the best of our knowledge, this is the first report of a patient with MBD that mimicked MND at clinical presentation.

## Conclusion

In conclusion, MBD can mimic MND, and physicians should include MBD in the differential diagnosis for patients with progressive dysarthria and dysphagia and motor weakness.

### Patient consent

Written informed consent was obtained from the patient for publication of this case report and any accompanying images. A copy of the written consent is available for review by the Editor-in-Chief of this journal.

## Abbreviations

MBD: Marchiafava-Bignami disease; FLAIR: Fluid attenuated inversion recovery; DWI: Diffusion-weighted image; MND: Motor neuron disease; ALS: Amyotrophic lateral sclerosis; MMSE: Mini-mental status examination; MMT: Manual muscle test.

## Competing interest

The authors declare that they have no competing of interest.

## Authors’ contributions

Acquisition of data: YH and YU. Analysis and interpretation of data: YH, YU, HS, NM, and MW. Drafting of the manuscript: YH, YU, HS, and TU. Critical revision of the manuscript for important intellectual content: YU, NH, and TU. All authors read and approved the final manuscript.

## Pre-publication history

The pre-publication history for this paper can be accessed here:

http://www.biomedcentral.com/1471-2377/13/208/prepub
